# Utility of erector spinae plane block in thoracic surgery

**DOI:** 10.1186/s13019-020-01118-x

**Published:** 2020-05-12

**Authors:** Naghmeh Pirsaharkhiz, Kelly Comolli, Wakana Fujiwara, Susan Stasiewicz, Jeanne M. Boyer, Eileen V. Begin, Adam J. Rubinstein, Hayley R. Henderson, John F. Lazar, Thomas J. Watson, Christopher M. Eger, Christine T. Trankiem, Debra G. Phillips, Puja Gaur Khaitan

**Affiliations:** 1 Department of General Surgery, Georgetown University School of Medicine, Medstar Washington Hospital Center, Washington DC, USA; 2 Department of Anesthesia, Medstar Washington Hospital Center, Washington DC, USA; 3 Department of Surgery, Division of Thoracic Surgery, Georgetown University School of Medicine, Medstar Washington Hospital Center, 110 Irving Street, NW (G253), Washington DC, 20010 USA; 4 Department of General Surgery, Division of Trauma, Medstar Washington Hospital Center, Washington DC, USA

**Keywords:** Erector spinae plane block; regional block, ERAS protocols, Thoracic surgery

## Abstract

**Background:**

Thoracic surgeons have been incorporating enhanced recovery after surgery (ERAS) protocols into their practices, not only to reduce narcotic usage but also to improve complication rates and decrease lengths of stay. Here, we describe the utility of a regional block technique that can be used for patients undergoing urgent or elective thoracic surgical procedures or suffering from rib fractures.

**Methods:**

We report our initial one-year experience with these erector spinae plane (ESP) blocks.

**Results:**

ESP blocks were placed in 42 patients. The procedure was performed by a trained team of anesthesiologists and certified nurse practitioners. It included placement of a catheter on the ipsilateral chest, followed by a 20 ml of 0.2% ropivacaine bolus and continuous infusion. Patients were then followed by the regional team, as long as the catheter was in place. While it had some technical challenges, the block was effective in 83.3% of patients with no reported mortality or major complications. However, given the confounding factors of the study (such as simultaneous implementation of ERAS protocol) and heterogeneity of the patient population, a control group was difficult to ascertain and meaningful opioid consumption analysis was difficult to perform.

**Conclusions:**

Regional blocks, such as the ESP block, complement fundamental ERAS principles and serve as an adjunct to the available armamentarium for non-narcotic ways to control pain in thoracic surgical and chest trauma patients. Continued collaboration between the thoracic surgeons and anesthesiologists is needed for its success.

## Background

Injury to the chest wall, whether from operative intervention or trauma, has the potential to cause significant pain. Adequate analgesia while recovering from chest surgery or trauma is essential to providing patient comfort and preventing complications, such as pneumonia or respiratory failure. Opioids, administered enterally or parenterally, have been the traditional mainstays of analgesic regimens for thoracic pain, though are associated with a number of undesirable side effects and the potential for both tolerance and addiction. In an era when a national “opioid crisis” has reached epidemic proportions in the United States, non-narcotic adjuncts to pain management have assumed increasing importance.

Thoracic surgeons, like practitioners in other disciplines, have been incorporating enhanced recovery after surgery (ERAS) protocols into their practices. Regional blocks offer advantages for postoperative pain control compared to other strategies, complementing fundamental ERAS methods and reducing the utilization of opioid medications. The erector spinae plane (ESP) block, first described by anesthesiologist Mauricio Forero in 2016, is a multidermatomal sensory block that provides regional anesthesia to the ipsilateral chest wall [1]. The sites of action are the dorsal and ventral rami of the thoracic spinal nerves, typically extending from the level of T3 to T10. Placement of the ESP block involves ultrasound-guided injection of a long-acting local anesthetic, commonly ropivacaine, between the erector spinae muscle and transverse spinal processes. The initial bolus can be followed by placement of an indwelling catheter to allow for prolonged continuous infusion.

The ESP block is an effective approach for analgesia in thoracic surgical and chest trauma patients, providing excellent pain relief while reducing narcotic requirements. Data on efficacy of pain control with ESP blockade is rapidly maturing with randomized controlled trials [2–5]. While much has been written on this subject in the anesthesiology literature including a recently pooled analysis [6–9], little has been published in the surgical journals [5, 10, 11]. This study assesses our initial one-year experience with ESP blocks on a thoracic surgical service at a large urban hospital.

## Methods

The patients in this study all were treated at an 850-bed academic teaching hospital (MedStar Washington Hospital Center) in the District of Columbia. Beginning in January 2018, the thoracic surgeons, trauma surgeons, and dedicated thoracic anesthesiologists collaborated to perform ESP blocks on patients undergoing minimally invasive and open thoracic surgical procedures, as well as on non-surgical patients recovering from blunt thoracic trauma resulting in rib fractures. The blocks were performed by members of the regional block team comprised of anesthesiologists and certified nurse practitioners. A retrospective analysis of all patients undergoing ESP blocks between January 1 and December 31, 2018 was undertaken after institutional review board (IRB) approval was obtained.

### Technique

**Fig. 1:**
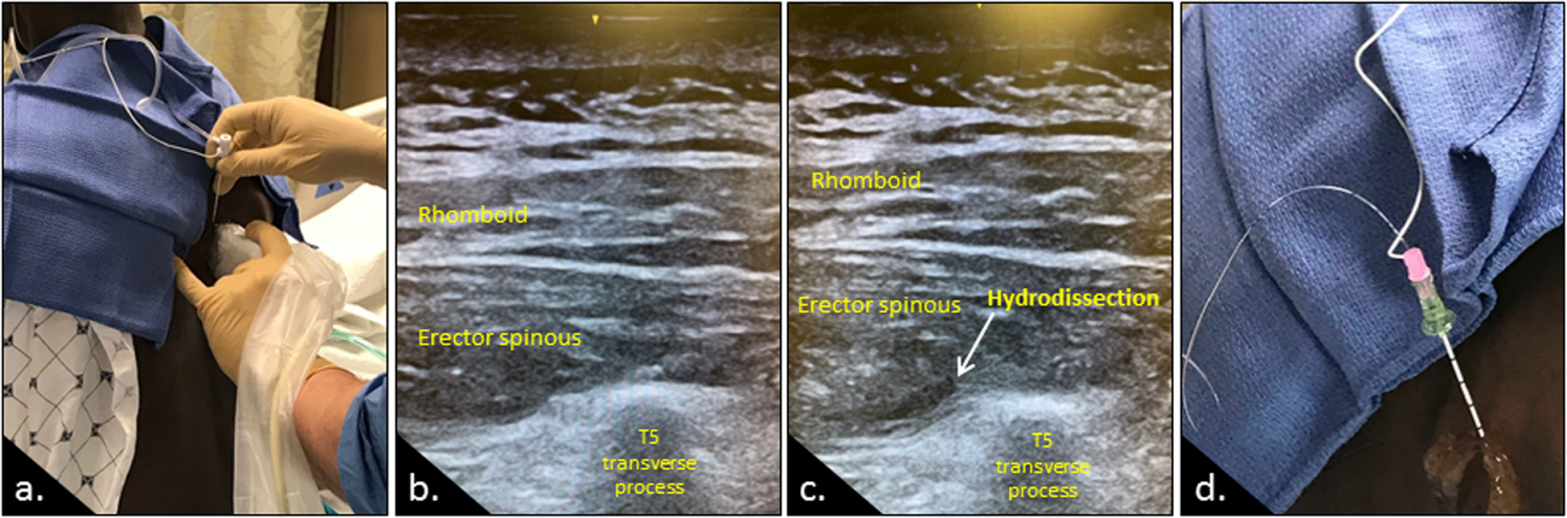
The erector spinae plane block (ESP) block is typically performed with the patient in an upright position in the preoperative holding area **a**. Using a 10 MHz ultrasound probe, the erector spine muscle is identified just above the T5 transverse process **b**. A plane (arrow) is developed deep to the muscle by injecting ropivacaine **c**. A wire is then threaded via the needle in this plane **d**, and confirmed to be in the plane on ultrasound prior to securing it

As seen in Fig. 1a, the blocks typically were performed (prior to the induction of general anesthesia in patients undergoing subsequent surgical procedures) with the patient upright and bent forward to allow the rib spaces to spread. Under sterile conditions, a 10 MHz ultrasound probe (GE LOGIQe, Wauwatosa, Wisconsin) was used to visualize the trapezius, rhomboid major, and erector spinae muscles approximately 3 cm lateral to the T5 spinous process on the target side (Fig. 1b). A 17-gauge Tuohy needle was advanced in a cephalad to caudad direction until the tip of the needle reached the plane deep to the erector spinae muscle and immediately lateral to the transverse process. A 20 ml bolus of the long-acting local anesthetic ropivacaine was injected in this plane, causing the erector spinae muscle to lift away from the transverse process and external intercostal muscle (Fig. 1c). An injection in this plane permitted the block to impact both the dorsal and ventral rami as they exited from the thoracic spine to innervate the chest wall. After a plane was developed, a catheter was introduced with multiple side-holes (Fig. 1d). The entire procedure took about 10 min, from setup to dressing. This catheter provided delivery of a continuous infusion once the bolus wore off, typically after a median of 12 h. The position of the catheter was confirmed via ultrasound at the end of the procedure, and the apparatus was secured to the chest wall. The block could be performed with the patient sitting (preferred) or in either lateral decubitus position, the latter of particular use if done at the end of an operation. The adequacy of the block was assessed using a pinprick test, with most patients reporting diminished sensation spanning the T3-T10 dermatome levels. Our protocol consisted of a continuous infusion of 0.2% ropivacaine at 10–12 ml/hour (following the initial bolus of 20 mL). All inpatients were followed on a daily basis by the anesthesiology regional pain service. The catheters were typically left in place between 2 and 14 days, based on clinical assessment.

## Results

Between January 1 and December 31, 2018, ESP blocks were performed in 42 patients undergoing thoracic surgical procedures or with rib fractures secondary to acute trauma. Patient demographics are detailed in Table 1. In general, urgent non-traumatic indications for ESP blockade included patients with non-emergent hemothoraces requiring decortication, chronic obstructive pulmonary disease (COPD) with blebs requiring blebectomy and pleurodesis, or with recurrent pleural effusions secondary to congestive heart failure (CHF) or end-stage renal disease (ESRD) requiring pleurodesis. Patients in the first category were usually post-cardiotomy or trauma patients who typically had a sternotomy or other injuries, whereas patients from the two latter categories typically had other comorbidities and some of the patients had been hospitalized for a while prior to surgical consultation for other disease processes. After a favorable preliminary experience with ESP blocks in trauma patients and those referred in-hospital with conditions in need of urgent intervention, we began to offer blocks to elective thoracic surgical patients, including those scheduled for minimally invasive lung resections. Of the 42 patients, 24 were elective cases.

**Table 1:** Patient demographics (n, 42)

Age (median)	59 (range, 27–82)
Gender	
Male	21
Indication	
Benign	26
Malignant	16
Procedure	
Minimally-invasive	
Robotic	12
Thoracoscopy	13
Thoracotomy	9*
Rib plating	4
Non-operative	4

* 4 out of 9 thoracotomies (44%) were muscle-sparing

While most patients experienced excellent pain relief, 7 of 42 (16.7%) did not benefit from their blocks. As seen in Table 2, the ESP catheter fell out in 3 of 42 patients, two on postoperative day (POD) 1 and another on POD 3. One patient was noted to have leakage around the catheter insertion site that resulted in removal on POD 1. Adequate pain control was not achieved in 3 additional patients. After retrospective evaluation, all 3 of these patients experienced technical issues during placement, as the operator was unable to develop the plane between the erector spinae muscle and external intercostal muscles and the catheter could not be advanced in the normal fashion. Importantly, no mortality or major complications, such as hematoma or neurologic deficit, resulted from ESP blockade.

**Table 2:** Complications directly related to ESP block

Minor Complications	
Catheter fell out	3
Leaking from catheter site	1
Technical failure	3
Major Complications	
Hematoma	0
Neurologic deficit	0
30-day mortality	0
90-day mortality	0

*One death occurred at 115 days due to severe heart failure

After excluding these 7 patients with ineffective blocks, we analyzed opioid consumption (measured as daily morphine equivalents during the time of ESP blockade) on the remaining 35 patients. On average the patients consumed 131.4 mg of morphine, with a median of 50.6 mg. Three patients required no opioids at all. This is in comparison to retrospective control patients with similar procedures who have not undergone any block in the same time-frame, where on average their consumption was as high as 1200 mg per our pharmacy statistics (data not shown, *p* < 0.005).

## Discussion

Regional anesthesia techniques, such as rectus sheath and transverse abdominis plane (TAP) blocks, are commonly applied for abdominal or pelvic surgeries and have multiple uses within the field of orthopedic surgery. Utilization of regional blocks has been associated with a reduction in postoperative narcotic requirements and associated adverse effects, such as ileus, constipation, delirium, or urinary retention that can lead to prolonged lengths of stay (LOS) in the hospital. In addition to the financial benefits associated with shorter LOS, regional anesthesia blocks lead to a quicker recovery and faster return to baseline levels of activity compared to completely enteral or parenteral narcotic pain management strategies [12–14]. The ESP block is a novel approach for post-operative and post-traumatic thoracic pain management. While much has been written about these blocks in the anesthesia and emergency medicine literature with success [6, 15], little has been published about them in the thoracic surgical literature [5, 10, 11]. We found ESP blockade to provide effective analgesia in the vast majority of patients following thoracic operations or trauma with acceptable risk, failure rates, and side effect profiles.

**Fig. 2:**
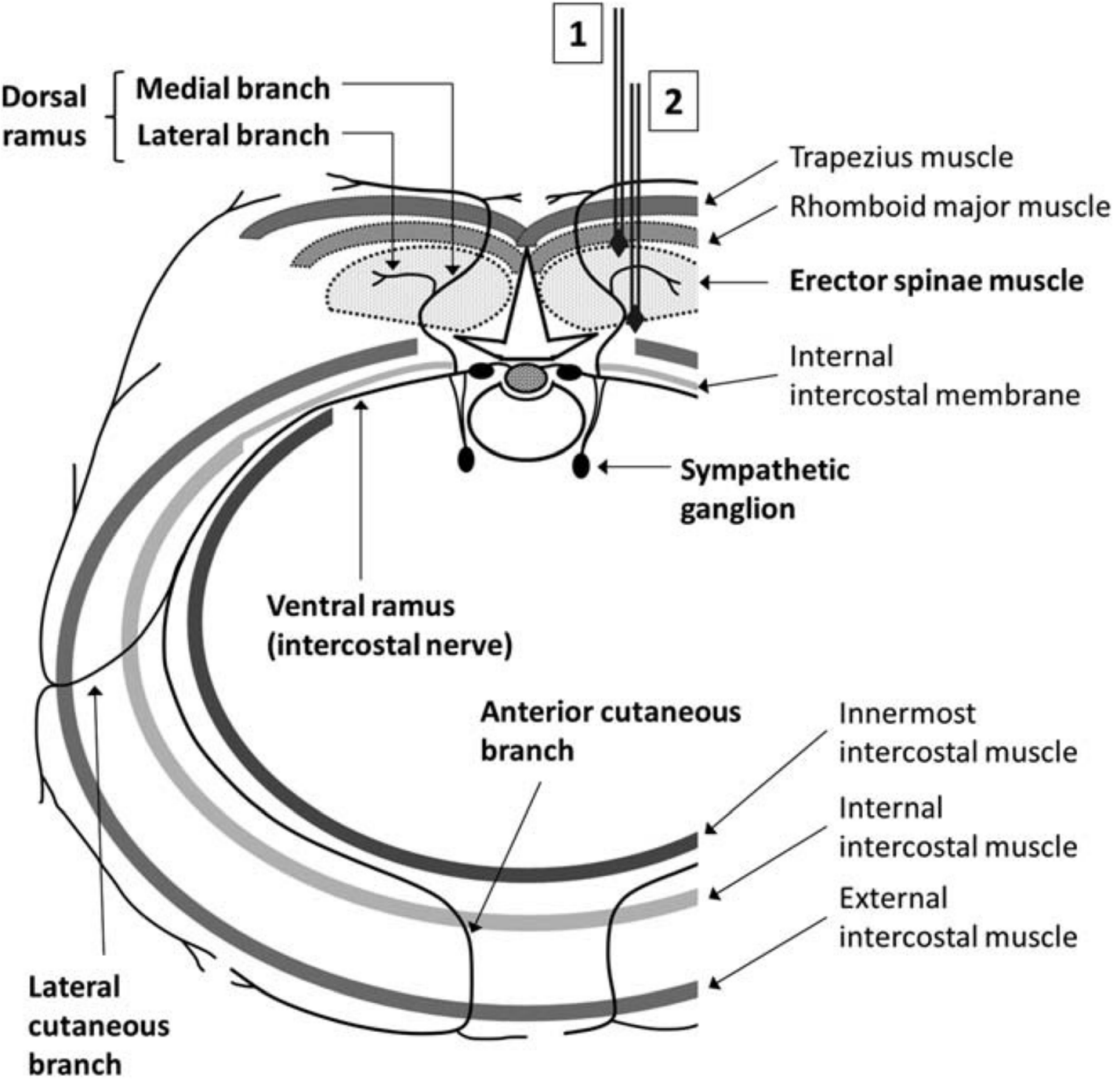
This schema illustrates the typical course of an upper thoracic spinal nerve. As the nerve exits the spinal foramen, it splits into the dorsal and ventral ramus. When an ESP block is placed appropriately (deep to the erector spinae muscle labeled as 2, vs. superficial to the ESP muscle labeled as 1), the block is able to affect both the dorsal and the ventral ramus. Unlike the ESP block, an intercostal nerve block only blocks the ventral ramus, but does not alleviate any sensory pain from the dorsal ramus. Written permission obtained from KJ Chin

Although other regional anesthesia blocks exist for use following thoracic surgical procedures, the novel ESP block provides unique advantages over previously described modalities. While thoracic surgeons can perform intercostal rib blocks intraoperatively by injection of a local anesthetic such as liposomal bupivacaine (Exparel; Pacira Pharmaceuticals, Inc. Parsippany, NJ), these blocks are not completely reliable and have low reproducibility. In a phase III clinical trial, liposomal bupivacaine did not achieve its primary endpoint of a reduction in cumulative pain scores at 72 h [16]. Several explanations have been hypothesized for the potential failure of intercostal nerve blocks. The local anesthetic may not reach the correct plane during each injection or the injectate may leak from the puncture sites. When injected intraoperatively from within the chest under direct visualization during open, video-assisted thoracoscopic surgery (VATS) or robotic-assisted thoracoscopic surgery (RATS) procedures, the parietal pleura is violated, allowing leakage within the pleural cavity. Consideration of the relevant anatomy would suggest that an intercostal block, unlike an ESP block, affects only the ventral ramus of the spinal nerve (Fig. 2), thereby leaving the dorsal ramus and crossing lateral cutaneous branches unaffected. The impact of intercostal blocks on pain emanating from cutaneous sources, therefore, is suspect. Finally, ESP blocks provide a potentially longer duration of analgesia delivery compared to intercostal rib blocks due to the placement of an indwelling analgesic catheter.

The ESP block technique is felt to be safer than that of both epidural and paravertebral blocks, as injection and catheter placement rarely are associated with adverse events such as epidural hematoma, headaches, urinary retention, or hypotension that can result from medication administration to the neuroaxis. Additionally, since ESP is considered a muscle or fascial planar block, it may be a safer alternative for patients with coagulation defects who otherwise might not be candidates for neuraxial blockade. And finally, the ESP block does not pose the risk of direct neurologic injury and, consequently, can be placed while the patient is anesthetized. Of note, we observed no serious complications in our series. Finally, recent prospective studies evaluating the efficacy of epidural catheters have reported a failure rate as high as 23–25% [17, 18].

The ESP block is part of a multimodal analgesia regimen that has the potential to impact postoperative pain as well as opioid requirements following thoracic surgical procedures. As more institutions adopt ERAS protocols into their surgical practices [19–21], neural blockade serves as a useful adjunct to those clinical care pathways in an effort to decrease narcotic use and promote faster recovery. The ESP blocks require a multidisciplinary team that includes thoracic/trauma surgeons, anesthesiologists, regional pain specialists, and nursing staff. In addition to its utility for elective thoracic surgical cases, the block adds a tool to the armamentarium for patients with rib fractures and post-traumatic thoracic pain.

Our study has several limitations. We did not perform a robust analysis of patients’ pain scores or comparisons to patients undergoing epidural, intercostal or no nerve blocks. Of the 42 patients enrolled in this study, some were inpatients, referred with urgent conditions such as pleural effusions resulting from CHF or ESRD, and about 57% underwent elective procedures. To compare patients undergoing VATS or RATS to patients undergoing a thoracotomy is somewhat disingenuous. As we were introducing the ESP block into our management strategy, we were simultaneously instituting an ERAS protocol for elective thoracic surgical procedures that included a 3-day preoperative course of analgesic medications (oral gabapentin, celecoxib, and acetaminophen) followed by a similar postoperative regimen. The relative impact of each of these components on postoperative pain relief could not be assessed. As a follow-up to the current study, we plan to investigate patients undergoing elective surgery who received an ESP block, and compare their postoperative outcomes to those receiving no block, in a case-control matched analysis where the type of surgery, surgical approach (minimally invasive or open), and the use of an ERAS protocol will be controlled between the two groups.

Finally, consistent with the performance of other types of blocks, there appears to be a learning curve for ESP blockade. Three patients in our series did not achieve adequate analgesia after ESP catheter placement was unsuccessful. In all 3 of these cases, the anesthesiologist attempting the blockade was inexperienced with the technique. While we suspect proper placement might have been achieved with more operator experience, anatomic abnormalities prohibiting successful placement could not be excluded.

## Conclusions

In an era when opioid addiction and its attendant problems have reached epidemic proportions, non-narcotic methods to control acute and chronic pain resulting from thoracic trauma or operations on the chest have assumed increasing importance. While thoracic surgeons and anesthesiologists have utilized a number of techniques for relief of thoracic pain, the ESP block is safe, effective, and reliable, providing several advantages compared to other approaches. Accordingly, ESP blockade can be considered in any patient undergoing elective, urgent or emergent thoracic surgical intervention or suffering from thoracic trauma and adds to the available armamentarium of analgesic techniques.

## Data Availability

The datasets used and/or analysed during the current study are available from the corresponding author on reasonable request.

## Abbreviations

ERAS: Enhanced recovery after surgery
ERPs: Enhanced recovery pathways
ESP: Erector spinae plane
RATS: Robot-assisted thoracic surgery
VATS: Video-assisted thoracic surgery
